# Peptide transporter isoforms are discriminated by the fluorophore‐conjugated dipeptides β‐Ala‐ and d‐Ala‐Lys‐N‐7‐amino‐4‐methylcoumarin‐3‐acetic acid

**DOI:** 10.1002/phy2.165

**Published:** 2013-12-08

**Authors:** Gabor Kottra, Britta Spanier, Tiziano Verri, Hannelore Daniel

**Affiliations:** 1 ZIEL Research Center of Nutrition and Food Sciences, Abteilung Biochemie, Technische Universität München, Gregor‐Mendel‐Str. 2, Freising, D‐85350, Germany; 2 Laboratory of General Physiology, Department of Biological and Environmental Sciences and Technologies, University of Salento, via Provinciale Lecce‐Monteroni, Lecce, I‐73100, Italy

**Keywords:** *Caenorhabditis elegans*, di‐ and tripeptide, PEPT1, PEPT2, substrate specificity, transporter

## Abstract

Peptide transporters of the SLC15 family are classified by structure and function into PEPT1 (low‐affinity/high‐capacity) and PEPT2 (high‐affinity/low‐capacity) isoforms. Despite the differences in kinetics, both transporter isoforms are reckoned to transport essentially all possible di‐ and tripeptides. We here report that the fluorophore‐conjugated dipeptide derivatives *β*‐Ala‐Lys‐N‐7‐amino‐4‐methylcoumarin‐3‐acetic acid (*β*‐AK‐AMCA) and d‐Ala‐Lys‐N‐7‐amino‐4‐methylcoumarin‐3‐acetic acid (d‐AK‐AMCA) are transported by distinct PEPT isoforms in a species‐specific manner. Transport of the fluorophore peptides was studied (1) in vitro after heterologous expression in *Xenopus* oocytes of PEPT1 and PEPT2 isoforms from different vertebrate species and of PEPT1 and PEPT2 transporters from *Caenorhabditis elegans* by using electrophysiological and fluorescence methods and (2) in vivo in *C. elegans* by using fluorescence methods. Our results indicate that both substrates are transported by the vertebrate “renal‐type” and the *C. elegans* “intestinal‐type” peptide transporter only. A systematic analysis among species finds four predicted amino acid residues along the sequence that may account for the substrate uptake differences observed between the vertebrate PEPT1/nematode PEPT2 and the vertebrate PEPT2/nematode PEPT1 subtype. This selectivity on basis of isoforms and species may be helpful in better defining the structure–function determinants of the proteins of the SLC15 family.

## Abstract (precis)

Peptide transporters of the SLC15 family can be classified by structure and function into the PEPT1 (low‐affinity/high‐capacity) and PEPT2 (high‐affinity/low‐capacity) phenotype. We found that the fluorophore‐conjugated dipeptide derivatives *β*‐Ala‐Lys‐N‐7‐amino‐4‐methylcoumarin‐3‐acetic acid (*β*‐AK‐AMCA) and d‐Ala‐Lys‐N‐7‐amino‐4‐methylcoumarin‐3‐acetic acid (d‐AK‐AMCA) are transported only by distinct PEPT isoforms in a species‐specific manner. This selectivity on basis of isoforms and species should be helpful in further defining the substrate‐binding domain of peptide transporters.

## Introduction

Fluorescent substrates or fluorophores attached to transport substrates have been used to observe and to quantify transport activity of various transporters (Bednarczyk et al. 2000; Schwartz et al. 2003; Mason et al. 2005; Brunaldi et al. 2007; Jorgensen et al. 2008). Peptide transporters of the SLC15 family are found in all organisms and primarily mediate the uptake of amino acids in di‐ and tripeptide form. Peptide transport over the plasma membrane is coupled to symport of protons and allows transport of over 8000 different di‐ and tripeptides against a substrate gradient. To avoid acidification of the cells, protons are exported in exchange with Na^+^ by the sodium‐proton exchanger NHE3, which explains the requirement of NHE3 in the enterocytes for PEPT1 function (Chen et al. 2010). Next to their natural substrates, peptide transporters also transport peptidomimetics such as aminocephalosporins, selected inhibitors of the angiotensin‐converting enzyme as well as various prodrugs (Ganapathy et al. 1995; Zhu et al. 2000; Shu et al. 2001). Several studies have employed as well the fluorophore‐conjugated dipeptide derivative *β*‐Ala‐Lys‐N‐7‐amino‐4‐methylcoumarin‐3‐acetic acid (*β*‐AK‐AMCA) to visualize and quantify transport activity of SLC15 peptide transporters. These include characterization of transport in LLC‐PK1 cells, rat kidney tubules, mammalian enteric and central nervous system cells and rat thyroid follicular cells that all were shown to express the high‐affinity peptide transporter PEPT2 (Wenzel et al. 1998; Dieck et al. 1999; Groneberg et al. 2002b, 2004; Rühl et al. 2005; Romano et al. 2010). *Caenorhabditis elegans* intestinal cells expressing cePEPT1 (Meissner et al. 2004) and *Escherichia coli* expressing YdgR (Weitz et al. 2007), both low‐affinity PEPT1‐like transporters, also show effective uptake of the fluorophore‐conjugated substrates, although both have only 35% (cePEPT1) and 22% (YdgR) identity when compared to human PEPT1. Transport of d‐Ala‐Lys‐N‐7‐amino‐4‐methylcoumarin‐3‐acetic acid (d‐AK‐AMCA) via PEPT1 into murine intestinal cells has also been reported previously (Groneberg et al. 2001). However, we were unable to demonstrate this in the intestine of wild‐type C57/BL6 mice in comparison to mice lacking PEPT1 (Pept1^−/−^). Neither d‐AK‐AMCA, nor *β*‐AK‐AMCA revealed a PEPT1‐specific transport into mouse intestinal cells. This prompted us to systematically investigate the transport characteristics of *β*‐AK‐AMCA and d‐AK‐AMCA in *Xenopus* oocytes expressing vertebrate PEPT1‐ and PEPT2‐type transporters and the *C. elegans* PEPT1‐ and PEPT2‐like proteins using the two‐electrode voltage clamp system. In addition, analysis of fluorescence in oocytes exposed to *β*‐AK‐AMCA was quantified in the absence and presence of the dipeptide glycyl‐glutamine (GQ), a reference substrate for both peptide transporter isoforms that acts as a competitor and namely competes with *β*‐AK‐AMCA in a concentration‐dependent manner. To assess whether the fluorophore unit attached to the *ε*‐aminogroup of the C‐terminal lysine discriminates for transport, uptake kinetics of the dipeptide *β*‐Ala‐Lys (*β*‐AK) was compared to that of *β*‐AK‐AMCA.

## Material and Methods

### Oocyte preparation

*Xenopus laevis* oocytes were collected under anesthesia (immersion in a solution of 0.7 g/L of 3‐aminobenzoic acid ethyl esther; Sigma, Steinheim, Germany) from frogs that were killed with an anesthetic overdose after the final oocyte collection. Oocytes were treated with 2.5 mg/mL collagenase in Barth's solution for 70 min and were separated manually thereafter. The selected oocytes were incubated in Barth's solution containing 88 mmol/L NaCl, 1 mmol/L KCl, 0.8 mmol/L MgSO_4_, 0.4 mmol/L CaCl_2_, 0.3 mmol/L Ca(NO_3_)_2_, 2.4 mmol/L NaHCO_3_, and 10 mmol/L HEPES (pH 7.5) at 17°C overnight.

### Transporter isoforms used

Human (hPEPT1; Liang et al. 1995), rabbit (rPEPT1; Boll et al. 1994), mouse (mPEPT1; Fei et al. 2000), zebrafish (zfPEPT1; Verri et al. 2003), and *C. elegans* (cePEPT1, formerly OPT‐2 or PEP‐2; Fei et al. 1998) PEPT1‐like, human (hPEPT2; Liu et al. 1995), rabbit (rPEPT2; Boll et al. 1996), zebrafish (zfPEPT2; Romano et al. 2006), and *C. elegans* (cePEPT2, formerly OPT‐1 or PEP‐1; Fei et al. 1998) PEPT2‐like transporters were used. cRNA from sequenced cDNA of all corresponding genes was synthetized using the mMESSAGE mMACHINE T7 kit (Ambion, Life Technologies, Darmstadt, Germany). Stage V/VI oocytes were injected with about 25 ng of PEPT1 or PEPT2 cRNA in 18–27 nL volume and incubated for 3–5 days in Barth's solution at 17°C. The amount of injected cRNA varied slightly between species (*C. elegans*, human, rabbit, zebrafish) reflecting different concentrations in the injection solution. The rabbit chimeric transporters rPEPT1/2 and rPEPT2/1 were constructed as described previously (Doring et al. 1996, 2002) and contained the first 59 N‐terminal amino acids from rPEPT1 and the amino acids 89–729 from rPEPT2 (rPEPT1/2) or the first 401 N‐terminal amino acids from rPEPT2 and the amino acids 382–707 from rPEPT1 (rPEPT2/1), respectively.

### Electrophysiology

Two‐electrode voltage clamp experiments were performed as described previously (Kottra and Daniel 2001). Briefly, the oocyte was placed in an open chamber (~0.5 mL total volume) and was continuously superfused (~3 mL/min) with Barth's solution in which HEPES was replaced by MES (for pH 6.5). Oocytes were voltage‐clamped at −60 mV using a TEC‐05 amplifier (NPI Electronic, Tamm, Germany) and current–voltage (I/V) relations were measured using short (100 msec) pulses separated by 200 msec pauses in the potential range −160 mV to +80 mV. I/V measurements were made immediately before and 20–30 sec after substrate application (including *β*‐AK‐AMCA, d‐AK‐AMCA, *β*‐AK, and GQ) when current flow reached steady state. The current evoked by the tested isoforms at a given membrane potential was calculated as the difference between the currents measured in the presence and absence of substrate. Substrate affinities were determined by measuring currents in the concentration range 0.5–4 mmol/L for cePEPT1 and 0.05–2 mmol/L for rPEPT2 and zfPEPT2, respectively, and by fitting the data to the Michaelis–Menten equation.

### Fluorescence measurements in oocytes

Forty to fifty oocytes per experimental group expressing the transporter to be tested were placed in single wells of a 24‐well plate. They were incubated in 0.5 mL Barth's solution containing 1 mmol/L *β*‐AK‐AMCA without or with 20 mmol/L GQ at pH 6.5 for 3 h in dark at 18°C. Control groups consisted of cRNA‐injected oocytes incubated in Barth's solution at pH 6.5 without *β*‐AK‐AMCA and of water‐injected oocytes incubated in Barth's solution with substrate. After incubation, oocytes were washed three times with Barth's solution, and after supernatant removal, homogenized by a Pellet Pestle on ice. Samples were centrifuged (15,500*g*) for 10 min at 4°C, and fluorescence of 25 *μ*L of the supernatant was measured in black 384‐well microplates at 445 nm at an excitation wavelength of 349 nm in a plate reader (Thermo Fischer Scientific, Waltham, MA). The experiments were repeated on oocytes originating from at least three different frogs and the means and standard error of means (SEM) were calculated.

### Fluorescence measurements in *C. elegans*

The following *C. elegans* strains were used: wild‐type N2 (var. Bristol) and cePEPT1 knockout (*pept‐1*(*lg601)X*; BR2742). The *C. elegans* strains were grown at 20°C as a mixed‐stage population on nematode growth medium (NGM) agar plates seeded with the food bacteria *E. coli* OP50 (Wood 1988). To synchronize populations, the mixed‐stage worm culture was washed off the plates with M9 buffer (22 mmol/L KH_2_PO_4_, 38.5 mmol/L Na_2_HPO_4_, 85.5 mmol/L NaCl, 1 mmol/L MgSO_4_) and eggs were prepared by hypochloride treatment. Synchronized L1 larvae were grown on NGM agar plates with *E. coli* OP50 as food source till the fourth larval state (L4). Synchronized L4 larvae were washed off the plates with M9 buffer. A final concentration of 1 mmol/L *β*‐AK‐AMCA or d‐AK‐AMCA was added to the buffer and the samples were incubated in an over‐head shaker for 10 min, 1 h, 2.5 h, and 5 h at 20°C. Worms were washed with M9 buffer and a sample was loaded to an object slide for visualization of the *β*‐AK‐AMCA or d‐AK‐AMCA uptake. A Leica TCS SP2 Confocal System with an UV laser coupled to a DM IRB microscope (Leica, Wetzlar, Germany) was used.

### Chemicals

*β*‐AK‐AMCA and d‐AK‐AMCA were from Bio Trend (Zurich, Switzerland). GQ was a generous gift by Degussa–Rexim (Hanau, Germany). All other substances were obtained from Sigma (Germany).

### Statistics

All values are expressed as means ± SEM for “*n*” independent measurements. Statistical differences were calculated with Student's *t* test for paired or unpaired samples as appropriate and were regarded as significant when *P* < 0.05.

## Results

### Electrophysiological transport measurements

**Figure 1.**
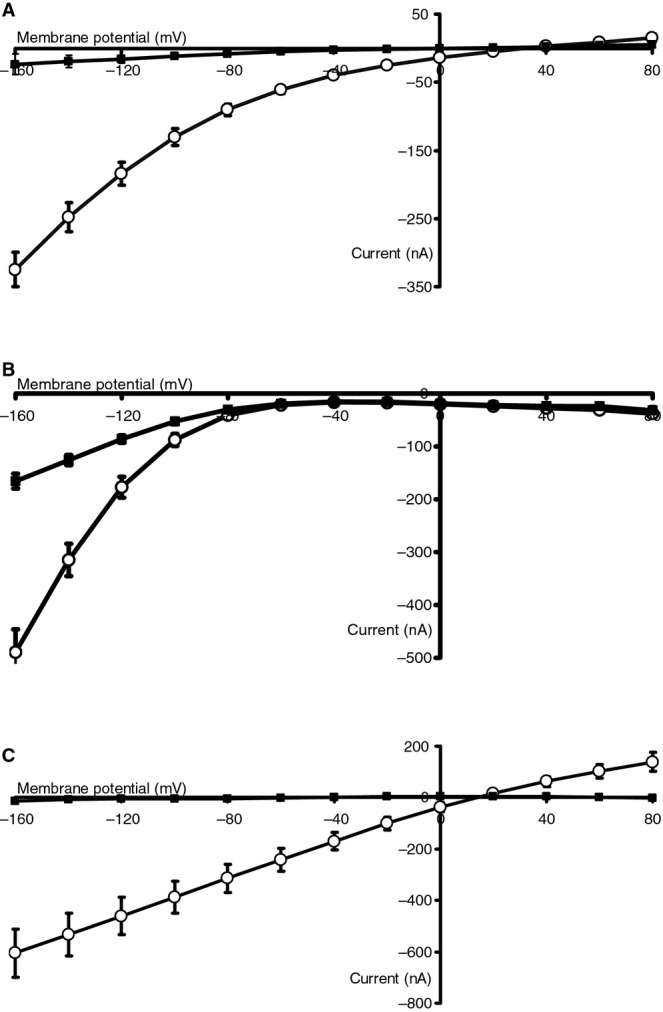
Current–voltage (I/V) relations induced by 1 mmol/L GQ (open circles) and by 1 mmol/L *β*‐AK‐AMCA (filled squares) in oocytes expressing (A) *Caenorhabditis elegans *PEPT1 (cePEPT1), (B) rabbit PEPT2 (rPEPT2), or (C) rabbit PEPT1 (rPEPT1). Please note the low, but significant transport current induced by *β*‐AK‐AMCA in cePEPT1, the higher current in rPEPT2 and the absence of *β*‐AK‐AMCA‐induced current in rPEPT1. Similar I/V relations have also been recorded in the other transporters listed in Table 1 (not shown).

Figure 1 shows the current–voltage relations of transport of 1 mmol/L *β*‐AK‐AMCA or 1 mmol/L GQ in oocytes expressing cePEPT1, rPEPT1, or rPEPT2. In these representative setups, *β*‐AK‐AMCA is transported by cePEPT1 and rPEPT2 but not by rPEPT1. Table 1 summarizes in a complete data set the mean transport currents induced by 1 mmol/L *β*‐AK‐AMCA and by 1 mmol/L d‐AK‐AMCA as compared to the currents induced by 1 mmol/L GQ in oocytes expressing cePEPT1, cePEPT2, hPEPT1, hPEPT2, rPEPT1, rPEPT2, mPEPT1 and by 1 mmol/L *β*‐AK‐AMCA in oocytes expressing zfPEPT1, zfPEPT2 and the chimeric transporters rPEPT1/2 and rPEPT2/1. The chimeric transporters were used to distinguish between the phenotypic characteristics of the more PEPT1‐like form with the first 59 N‐terminal amino acids derived from rPEPT1 replacing the corresponding region in rPEPT2 in the chimera rPEPT1/2 (Doring et al. 2002), and the more PEPT2‐like form with the first 401 N‐terminal amino acids of rPEPT2 replacing the corresponding region in rPEPT1 in the chimera rPEPT2/1 (Doring et al. 1996). The transport of the reference substrate GQ in all groups (see Table 1) indicates the proper function of all PEPT isoforms in the heterologous expression system. Significant transport currents with the substrates *β*‐AK‐AMCA and d‐AK‐AMCA were observed only in cePEPT1, hPEPT2, rPEPT2, zfPEPT2, mPEPT1, and rPEPT2/1 expressing oocytes. Although hPEPT1, rPEPT1, zfPEPT1, mPEPT1, and rPEPT1/2 transported efficiently GQ, neither *β*‐AK‐AMCA nor d‐AK‐AMCA generated any, and in the case of mPEPT1, minimal currents, whereas only one of three cePEPT2 expressing oocytes generated measurable transport current even in the presence of high concentration of GQ. The apparent affinity of *β*‐AK‐AMCA for transport via cePEPT1 was *K*_m_ = 4.64 mmol/L and thus similar to that reported for glycyl‐sarcosine transport in *C. elegans* (Fei et al. 1998). As expected, rPEPT2 and zfPEPT2 revealed apparent affinities for *β*‐AK‐AMCA that were much higher with *K*_m_ values in the submillimolar range, but still lower than that of GQ. Extremely low currents of the chimeric rPEPT2/1 protein did not allow affinity to be determined. To assess whether the missing or the minimal transport currents in hPEPT1, rPEPT1, mPEPT1, and zfPEPT1 were caused by the fluorophore unit, we also determined uptake of the parent substrate *β*‐AK. Although transport currents were much lower than those of GQ in the same oocytes, these four transporter isoforms generated significant transport currents with this dipeptide (Table 1). Relative currents (in relation to GQ) generated by *β*‐AK in hPEPT2, rPEPT2, and zfPEPT2 were considerably higher than currents observed in the intestinal‐type transporters, but were generally lower than the currents generated by *β*‐AK‐AMCA.

**Table 1.** Transport currents generated by 1 mmol/L *β*‐AK‐AMCA,* β*‐AK and d‐AK‐AMCA as percent of current produced by the reference substrate (GQ) in oocytes.

	cePEPT1 (*n* = 6–16)	cePEPT2 (*n* = 3)	hPEPT1 (*n* = 6–8)	hPEPT2 (*n* = 5)	rPEPT1 (*n* = 6–10)	rPEPT2 (*n* = 5–11)	zfPEPT1 (*n* = 6–8)	zfPEPT2 (*n* = 4–13)	mPEPT1 (*n* = 6–8)	rPEPT1/2 (*n* = 3)	rPEPT2/1 (*n* = 4)
GQ (nA)	53.8	27.1	341.7	318.4	241.7	489.4	98.2	590.0	874.0	54.1	22.9
SEM	7.9	22.4	66.8	147.6	44.1	43.3	15.1	65.5	82	5.5	5.2
*β*‐AK‐AMCA vs. GQ	+ 18.9%	n.d.	1.8%	++ 42.1%	1.5%	++ 35.5%	−0.9%	++ 38.9%	+ 4.0%	6.5%	++ 5.7%
SEM	6.8%		1.4%	7.3%	0.9%	3.9%	0.5%	1.3%	1.2%	11.9%	1.3%
app. *K*_m_ *β*‐AK‐AMCA (mmol/L)	4.64	n.d.	n.d.	n.d.	n.d.	0.290	n.d.	0.296	n.d.	n.d.	n.d.
SEM (mmol/L)	1.67					0.027		0.026			
app. *K*_m_ GQ (mmol/L)	7.45	n.d.	n.d.	n.d.	n.d.	0.150	n.d.	0.079	n.d.	n.d.	n.d.
SEM (mmol/L)	0.61					0.033		0.012			
*β*‐AK vs. GQ	n.d.	n.d.	++ 7.0%	+ 27.1%	++ 11.6%	++ 21.0%	+ 7.9%	++ 19.2%	++ 10.1%	n.d.	n.d.
SEM			0.7%	6.7%	2.5%	2.2%	2.8%	2.1%	1.0%		
d‐AK‐AMCA vs. GQ	++ 13.8%	n.d.	−13.1%	++ 78.0%	1.0%	++ 44.5%	n.d.	n.d.	1.9%	n.d.	n.d.
SEM	2.0%		7.7%	17.9%	1.9%	10.1%			0.6%		

Due to the different current/voltage characteristics, currents for cePEPT1, hPEPT1, rPEPT1, mPEPT1, and zfPEPT1 as well as for the chimeric transporters (rPEPT1/2 and rPEPT2/1) are shown at −60 mV membrane potential, for cePEPT2, hPEPT2, rPEPT2, and zfPEPT2 at −160 mV. The currents induced by 1 mmol/L GQ are shown as absolute values, for the other substrates the relative values in percent of the GQ‐induced currents are shown. The *K*_m_ values for GQ are shown for comparison and were in part published previously. Significant currents are denoted by “+” (*P* < 5%) or “++” (*P* < 1%). n.d., not detectable.

### Fluorescence measurements in oocytes

**Figure 2.**
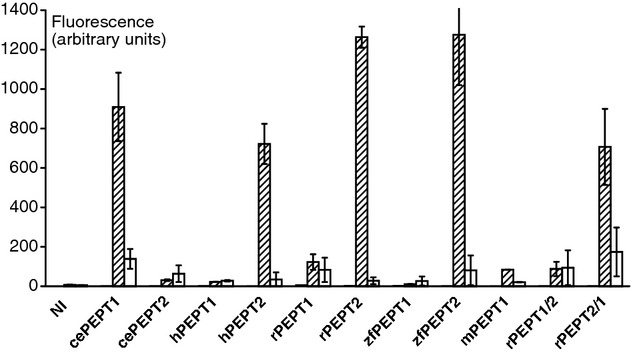
Fluorescence (arbitrary units) induced by 3 h incubation in solutions containing 1 mmol/L *β*‐AK‐AMCA without or with 20 mmol/L GQ in oocytes expressing *Caenorhabditis elegans* (ce), human (h), rabbit (r), zebrafish (zf), mouse (m) PEPT1 and PEPT2 transporters, and rabbit chimeric transporters. NI, non‐injected. Dark columns: without *β*‐AK‐AMCA; shaded columns: with 1 mmol/L *β*‐AK‐AMCA; open columns: with 1 mmol/L *β*‐AK‐AMCA + 20 mmol/L GQ. For details see Materials and Methods.

Figure 2 shows the results of fluorescence measurements in oocytes using the same transporter isoforms as in the electrophysiological studies. The endogenous fluorescence of either noninjected oocytes or oocytes injected with any transporter in the absence of the test compounds was very low. Exposure of oocytes to a solution containing 1 mmol/L *β*‐AK‐AMCA drastically increased fluorescence in oocytes expressing cePEPT1, hPEPT2, rPEPT2, zfPEPT2, and rPEPT2/1, which was in each case suppressed by the addition of an excess (20 mmol/L) of GQ. These data indicate the functional expression of the transporters in the heterologous expression system and confirm their substrate specificity by competition with the endogenous substrate GQ. In contrast, in oocytes expressing cePEPT2, hPEPT1, rPEPT1, zfPEPT1, or rPEPT1/2, fluorescence remained low after exposure to *β*‐AK‐AMCA in the absence or the presence of 20 mmol/L GQ.

### “In situ” fluorescence measurements in *C. elegans*

**Figure 3.**
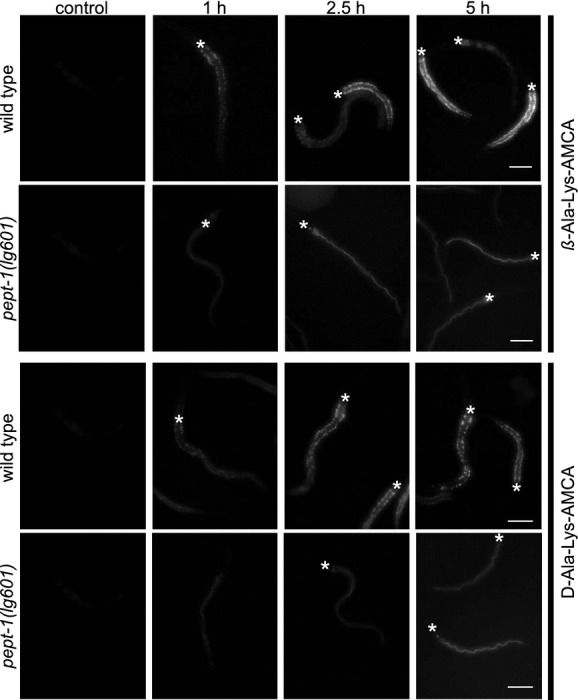
Time‐dependent uptake of *β*‐AK‐AMCA and d‐AK‐AMCA in wild type and peptide transporter deficient *pept‐1(lg601) Caenorhabditis elegans*. The animals were incubated for 10 min, 1 h, 2.5 h, and 5 h in medium containing 1 mmol/L *β*‐ or d‐AK‐AMCA and representative images are shown. The fluorescent signal is mostly detectable in the intestinal epithelial cells. The anterior end of the nematodes in the representative images is positioned to the top and the asterix mark the terminal bulb of the pharynx (*). Scale bars indicate 50 *μ*m.

Although we (Meissner et al. 2004), and others (Allman et al. 2009) have previously shown that *β*‐AK‐AMCA is transported in vivo by *C. elegans* cePEPT1, time‐dependent transport kinetics were not examined previously. When animals were exposed to 1 mmol/L *β*‐AK‐AMCA or d‐AK‐AMCA for 10 min or 60 min no significant absorption was detectable in wild‐type *C. elegans,* whereas exposure for 2.5 h led to an accumulation of the dye in the intestinal epithelial cells (Fig. 3). The fluorescence intensity increased slightly with an exposure time of 5 h in case of *β*‐AK‐AMCA, but not in case of d‐AK‐AMCA. Furthermore, uptake of both compounds was clearly dependent on cePEPT1, as in the corresponding knockout strain *pept‐1(lg601)* both labeled dipeptides accumulated in the intestinal lumen without any staining of epithelial cells. Together with our findings using the electrophysiological methods, we can conclude that *β*‐AK‐AMCA and d‐AK‐AMCA are transported *via* cePEPT1, but with low capacity.

### Sequence analysis of PEPT isoforms

**Figure 4.**
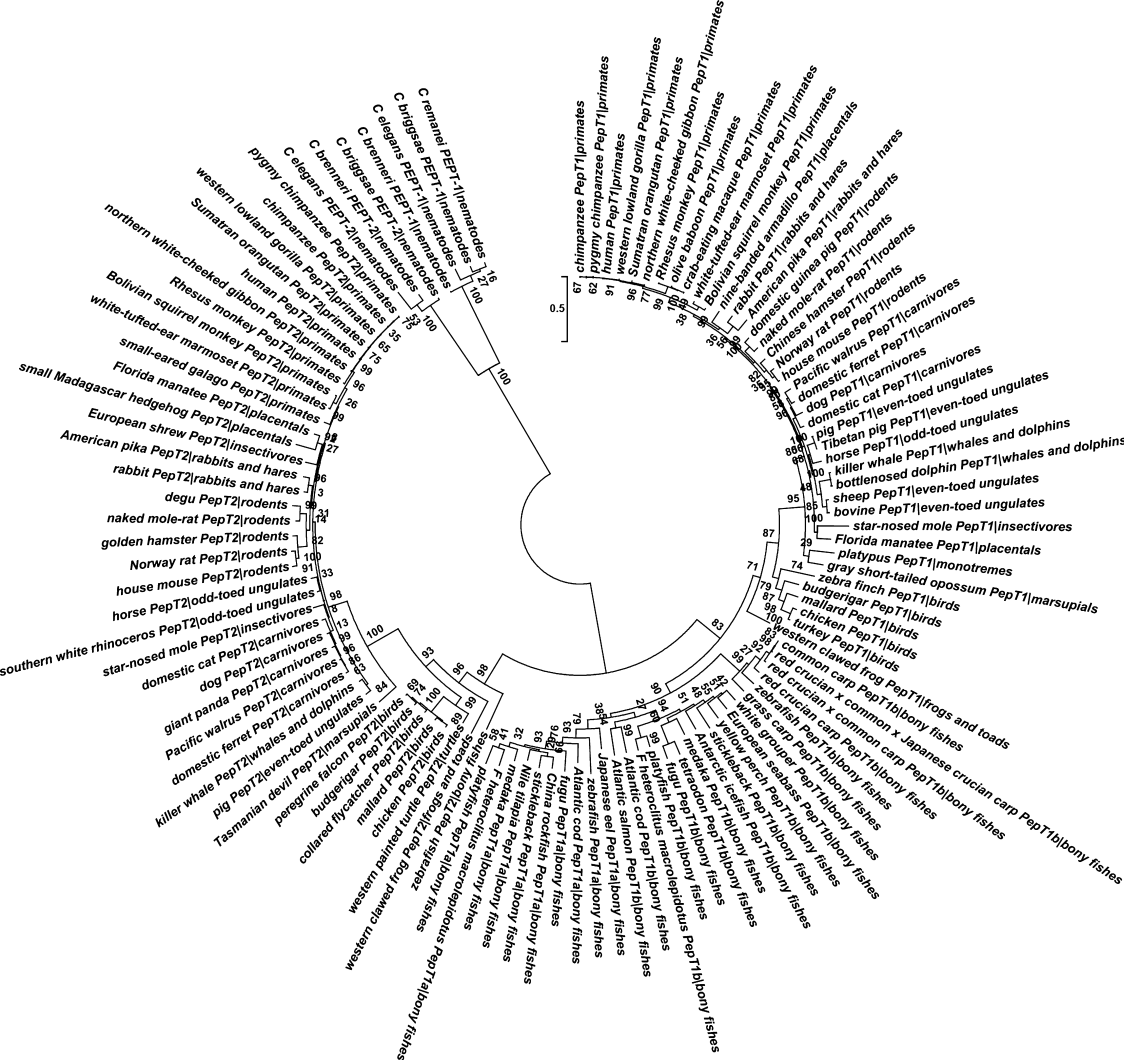
Molecular phylogenetic analysis performed on vertebrate PEPT1 and PEPT2 and nematode PEPT1 and PEPT2 by maximum likelihood (ML) method. The evolutionary history was inferred by using the ML method based on the Whelan and Goldman (2001) model. The tree with the highest log likelihood (−37,196.4871) is shown. Initial tree(s) for the heuristic search were obtained automatically as follows. When the number of common sites was <100 or less than one‐fourth of the total number of sites, the maximum parsimony method was used; otherwise BIONJ method with MCL distance matrix was used. A discrete Gamma distribution was used to model evolutionary rate differences among sites (five categories; +G, parameter = 0.9600). The rate variation model allowed for some sites to be evolutionarily invariable ([+I], 9.1163% sites). The tree is drawn to scale, with branch lengths measured in the number of substitutions per site. The analysis involved 114 amino acid sequences. All positions with less than 95% site coverage were eliminated. That is, fewer than 5% alignment gaps, missing data, and ambiguous bases were allowed at any position. There were a total of 630 positions in the final data set. Evolutionary analyses were conducted in MEGA5 (Tamura et al. 2011).

**Figure 5.**
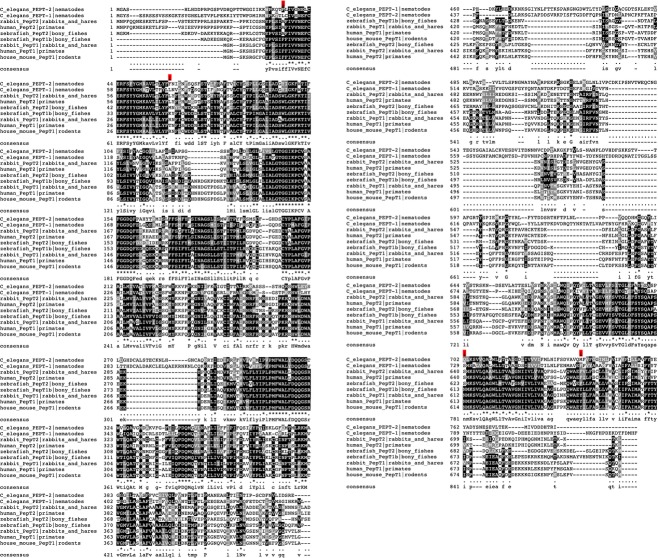
Sequence comparison performed on rabbit, human, mouse and zebrafish PEPT1, rabbit, human and zebrafish PEPT2, *Caenorhabditis elegans *PEPT1, and *C. elegans *PEPT2 amino acid sequences. Multiple sequence alignment was obtained by Clustal Omega (1.2.0) using default parameters. Consensus is reported. Candidate amino acid substitutions along the sequence are indicated with an arrow highlighted in red.

In the absence of any resolved structures from vertebrate and/or nematode peptide transporters it is not obvious to find out why subtle differences in protein responsiveness do exist among peptide transporter isoforms cloned from different species. In an attempt to answer why the nematode PEPT1‐type isoform behaves like vertebrate PEPT2 a comparative approach has been followed to possibly identify amino acids that may have been conserved during the evolution and that may account for the differences observed in the various vertebrate and nematode isoforms in presence of *β*‐AK‐ and d‐AK‐AMCA. First, the various clones were ordered based on their observed transport phenotypes (Table 2). When grouped on an evolutionary basis, the various clones could be categorized in the following phenotypes: mammalian PEPT2, zfPEPT2, mammalian PEPT1, zfPEPT1, nematode PEPT2, and nematode PEPT1. All isoforms transport GQ. However, mammalian PEPT2 proteins transport *β*‐AK, *β*‐AK‐AMCA, and d‐AK‐AMCA, whereas zfPEPT2 transports *β*‐AK and *β*‐AK‐AMCA but not d‐AK‐AMCA. On the other hand, mammalian PEPT1 proteins transport *β*‐AK well and *β*‐AK‐AMCA and d‐AK‐AMCA very slightly, whereas zfPEPT1 transports *β*‐AK well, *β*‐AK‐AMCA very slightly and d‐AK‐AMCA not at all (for details see Table 1). The differences in substrate specificity observed for the zebrafish proteins with respect to the mammalian counterparts are not unexpected (for a recent review see Romano et al. 2013). Second, an alignment among vertebrate PEPT1 and PEPT2 and nematode PEPT1 and PEPT2 amino acid sequences (114 sequences among PEPT1‐ and PEPT2‐type orthologs/paralogs) was performed by Clustal O (1.2.0) using default parameters (data not shown), with the phylogenetic tree depicted in Figure 4 summarizing the distances measured among those sequences. Then, based on the structure data from prokaryotic peptide transporters published by Newstead (2011), Solcan et al. (2012), Guettou et al. (2013), and Doki et al. (2013) those amino acids that had been found relevant for explaining the peptide transport mechanism were annotated. On that basis several amino acid residues could be grouped around the following major structural–functional elements of the transporter: extracellular gate, substrate‐binding site, and intracellular gate. Overall, although a somehow comparable sequence organization could be noticed between the N‐terminal half of nematode PEPT1/2 proteins and vertebrate PEPT2 transporters, no coherent combination of amino acid substitutions versus species‐specific transport phenotypes could clearly be drawn, although at least four residues might be put in correlation with the more vertebrate PEPT2‐like behavior of nematode PEPT1 (Fig. 5). In particular, at the N‐terminus amino acid position 17 in hPEPT1 is a phenylalanine (Phe17), which is also present in all vertebrate PEPT1 proteins and in nematode PEPT2 (Phe35), whereas a leucine or an alanine is present in vertebrate PEPT2 and in nematode PEPT1 (Leu49). Also, while position 42 presents a charged residue in hPEPT1 (Thr42), mPEPT1 (Arg42), rPEPT1 (Arg42), and zfPEPT1 (Lys49), a leucine or a methionine is present in vertebrate PEPT2 and in nematode PEPT1 (Leu74). Interestingly, this couple of residues is located at the beginning and at the end of helix H1 (one of the most conserved regions in these transporters), and encompass the crucial sequence motif ExxERFxYY – that is absolutely necessary for transport function – and a set of other residues highly conserved along the evolution. Moreover, at the C‐terminus amino acid position 613 in hPEPT1 is a serine (Ser613), which is also present in all vertebrate PEPT2 proteins and in nematode PEPT1 (Ser729), whereas an asparagine is present in vertebrate PEPT1 and a glutamine in nematode PEPT2 (Gln702). Also, in correspondence to position 649 in hPEPT1 is a tyrosine (Tyr649 in hPEPT1, Tyr650 in mPEPT1, Tyr648 in rPEPT1, and Tyr649 in zfPEPT1), whereas a phenylalanine is present in vertebrate PEPT2 and in nematode PEPT1 (Phe765), and a methionine in nematode PEPT2 (Met738). Interestingly, of this couple of residues one is located at the intracellular loop between helix H10 and helix H11, the other at the beginning of helix H12. So, one of these residues is located upstream, the other downstream a group of amino acids in helix H11 among which are a highly conserved glutamine (ref. Gln619 in hPEPT1), that has been identified as part of the intracellular gate in prokaryotic GkPOT, and a highly conserved tryptophan (ref. Trp622 in hPEPT1), that comes to be part of the substrate‐binding pocket. Whether these residues play a crucial role in determining the transport characteristics of the peptide transporters awaits further studies.

**Table 2.** Transport phenotypes exhibited by vertebrate (mammalian and zebrafish) and nematode proteins.

	Vertebrates							Invertebrates		Chimeric constructs	
	Mammalian PEPT2		Zebrafish PEPT2	Mammalian PEPT1			Zebrafish PEPT1	Nematode PEPT2	Nematode PEPT1		
	rPEPT2	hPEPT2	zfPEPT2	rPEPT1	hPEPT1	mPEPT1	zfPEPT1	cePEPT2	cePEPT1	rPEPT2/1	rPEPT1/2
GQ	+	+	+	+	+	+	+	+	+	+	+
*β*‐AK	+	+	+	+	+	+	+	−	−	−	−
*β*‐AK‐AMCA	+	+	+	+/−	+/−	+/−	+/−	−	+	+	+/−
d‐AK‐AMCA	+	+	−	+/−	+/−	+/−	−	−	+	−	−

For comparison, the transport phenotype of the two chimeric constructs is also reported (for details see the main body of the manuscript). +, transport detectable; +/−, slight transport detectable; −, no transport detectable.

## Discussion

Peptide transporters are members of the large group of solute membrane carriers SLC and are solely responsible for transport of more than 8000 different di‐ and tripeptides at epithelia from intestine, kidney, lung, and others. Due to their broad substrate spectrum they also transport drugs and peptidomimetics like *β*‐lactam antibiotics, some ACE inhibitors, protease inhibitors and antivirals. For visualization of these transport processes often fluorescence‐ or radiolabeled substrates are used. However, the coupling of relatively small peptides to bulky moieties might significantly alter their affinity to the transporter. Our results employing electrophysiological and fluorescence measurements in oocytes with various peptide transporter isoforms of different species clearly establish that the fluorophore‐conjugated dipeptides *β*‐AK‐AMCA and d‐AK‐AMCA are transported by the high‐affinity “renal‐type” vertebrate transporters and by the invertebrate cePEPT1 only. Neither mammalian PEPT1, nor zfPEPT1 or cePEPT2 show any significant transport of the labeled substrates when compared to the reference dipeptide GQ.

The fluorophore‐conjugated dipeptides used in this study carry either a *β*‐amino group (*β*‐AK‐AMCA) or a d‐amino acid (d‐AK‐AMCA) in N‐terminal position. Both alterations when compared with standard l‐*α*‐amino acids increase the stability of the peptides against hydrolysis by membrane‐bound and intracellular aminopeptidases and dipeptidases. However, the N‐terminus also plays a critical role in determining affinity for binding and transport by all peptide transporter isoforms. Peptide carriers generally prefer peptides with l‐*α*‐amino acid in N‐terminal position, but the magnitude of change in *K*_m_ also depends on other features of the substrate such as hydrophobicity/charge or bulkiness of the side chain and the overall charge of the substrate (Gebauer et al. 2003; Biegel et al. 2005; Larsen et al. 2008). Although a *β*‐ as compared to an *α*‐amino group reduces substrate affinity, transport of dipeptides such as *β*‐Ala‐Ala (Theis et al. 2002a), tripeptides like *β*‐Ala‐Gly‐Gly (Ganapathy et al. 1985) or carnosine (*β*‐alanyl‐l‐histidine) regularly occur, which has been useful in assessing some prime functions of PEPT1 and PEPT2 in various cell systems and tissues (Shu et al. 2001; Toyobuku et al. 2002; Son et al. 2004; Xiang et al. 2006). Although we here did not test the transport of d‐Ala‐Lys, its affinity for hPEPT1 has been determined previously (Brandsch et al. 2003) and was found to be considerably lower (app. *K*_m_ = 7.0 mmol/L) than that of the LL enantiomer l‐Ala‐Lys (app. *K*_m_ = 0.21 mmol/L). Such an affinity is still in the range of substrates of PEPT1 and similar to that observed here for *β*‐AK‐AMCA for transport *via* cePEPT1. Other dipeptides with d‐amino acid in N‐terminal position have also been shown to be transported by PEPT1, although with a lower affinity than the corresponding LL enantiomers, for example, d‐Ala‐l‐Ala (app. *K*_m_ = 0.80 mmol/L) versus l‐Ala‐l‐Ala (app. *K*_m_ = 0.16 mmol/L) (Daniel 2004) or d‐Ala‐l‐Pro (app. *K*_m_ = 5.0 mmol/L) versus l‐Ala‐l‐Pro (app. *K*_m_ = 0.15 mmol/L) (Brandsch et al. 2003).

These considerations all lead to the conclusion that the lack of transport of d‐AK‐AMCA or *β*‐AK‐AMCA by any of the PEPT1 isoforms must result from the attached fluorophore. When *β*‐AK was used as a substrate, all PEPT1 isoforms were indeed able to generate significant inward currents proving that not the modified N‐terminus but the bulky coumarin moiety prevents transport. It has been reported that the location and the size of side‐chain modifications critically controls transport differently in PEPT1 and PEPT2 and builds the basis for synthesis of high‐affinity inhibitors of the peptide transporters (Knutter et al. 2001; Theis et al. 2002b). In case of both transporters, a [Z(NO_2_)] group attached to the *ε*‐group of lysine in N‐terminal position as in Lys[Z(NO_2_)]‐Ala generated a high‐affinity inhibitor whereas when reversed as in Ala‐Lys[Z(NO_2_)] a high‐affinity substrate was obtained. Our present finding that a benzopyrone group such as coumarin placed in the same position prevents transport by vertebrate PEPT1 proteins but not by PEPT2 is the first example of discriminating substrates that allow transport by only one isoform. However, this lack of transport of fluorophore‐conjugated dipeptides *via* PEPT1 was also observed when either a coumarin or a fluoresceinisocyanate was attached to a C‐terminal *ε*‐aminogroup of lysine provided in a dipeptide such as Val‐Lys (Abe et al. 1999). Although these compounds revealed a remarkably high‐affinity for PEPT1 and could block transport of the model substrate glycyl‐sarcosine, they failed to be transported. However, PEPT2 isoforms seem to tolerate the bulky and hydrophobic side‐chain modifications quite well. In this respect, it is interesting to note that higher transport currents at identical concentration of *β*‐AK‐AMCA as compared to *β*‐AK were observed in all vertebrate “renal‐type” transporters in this study. A detailed study to identify those regions in the substrate structures that are responsible for recognition and for differences in affinity between PEPT1 and PEPT2 has been provided by Biegel et al. (2006). The disproportionally higher affinities for hydrophobic compounds observed for PEPT2 in contrast to PEPT1 were explained by considerably larger hydrophobic fields in the former transporter and this seems also a critical determinant in the capability of the PEPT2 isoforms to accept the fluorophore‐conjugated substrates employed here. Unfortunately, the very low transport currents of chimeric proteins of rabbit comprising the N‐terminal half of PEPT1 fused to the C‐terminal half of PEPT2 did not allow a more detailed analysis which protein domain discriminates or allows binding of the fluorophore‐coupled substrates.

A striking finding of the current analysis is that cePEPT1 as the only PEPT1 isoform of all tested species is able to generate transport currents with both fluorescent substrates. In case of *β*‐AK‐AMCA, the apparent affinity was even similar to that of the reference substrate GQ that assigns cePEPT1 to the low‐affinity PEPT1‐like class of the SLC15 family. The specificity for uptake of both fluorescent peptide derivatives via cePEPT1 in wild‐type animals is shown here by lack of uptake in animals deficient of PEPT1, which leads to the accumulation of substrates in the intestinal lumen. This finding, however, offers the opportunity to use *β*‐AK‐AMCA and d‐AK‐AMCA in a fine mapping of the substrate‐binding sites of chimeric proteins composed of *C. elegans* and mammalian proteins. What makes cePEPT1 in this respect different from all vertebrate PEPT1 transporters remains currently unexplained. However, a detailed sequence analysis of various PEPT isoforms suggested four residues that may account for the differences observed between the vertebrate PEPT1/nematode PEPT2 subtype and the vertebrate PEPT2/nematode PEPT1 subtype (for details see Results). Whether these residues play a crucial role in determining the transport characteristics of the peptide transporters awaits further studies.

Although the uptake of d‐AK‐AMCA via PEPT1 in murine enterocytes was reported before (Groneberg et al. 2001), we showed in this study that neither *β*‐AK‐AMCA nor d‐AK‐AMCA is a feasible substrate for mPEPT1 or any other vertebrate PEPT1 tested. These results are in accordance with data from rats systemically perfused with d‐AK‐AMCA that exhibited no accumulation of the fluorescent‐labeled dipeptide in enterocytes, but in the proximal tubules of kidney (Groneberg et al. 2002a). Furthermore, transport and uptake of *β*‐AK‐AMCA was only detectable in various tissues that express PEPT2 (Dieck et al. 1999; Groneberg et al. 2004; Rühl et al. 2005). For this purpose, we discourage from using *β*‐AK‐AMCA and d‐AK‐AMCA to detect PEPT1 transport function in enterocytes or intestinal‐like organoids differentiated from human induced pluripotent stem cells (iPS) as currently published (Spence et al. 2011; Iwao et al. 2013).

The practical consequence of our present findings is that *β*‐AK‐AMCA or d‐AK‐AMCA is not suitable to investigate transport properties in mammalian intestinal‐type transporters, whereas it can be used as a marker substrate for the intestinal peptide transport in *C. elegans*. The differences observed for the vertebrate and *C. elegans* transporters, suggests that in spite of numerous structural and functional similarities, distinct differences must exist in the substrate‐binding domains. Systematic structural–functional analysis of vertebrate versus nematode versus prokaryotic peptide transporters is needed to fully address why selective transport (or not transport) of a given peptide‐like molecule occurs in a given peptide transporter. The identification of such subtle differences might be of enormous help in defining the structural–functional rationale of substrate uptake in such diverse organisms with relevant impact in physiology, pathology, and pharmacology.

## Acknowledgments

The authors thank Daniela Kolmeder, who reconstructed the chimeric transporters and together with Katrin Lasch made the cRNA; Rainer Reichlmeir, who made a part of the electrophysiological experiments, and will be in our hearts and our minds forever; Helene Prunkl, who injected the oocytes and together with Beate Rauscher performed the fluorescence measurements; Jacqueline Benner, who did the uptake experiments in the *C. elegans*.

## Conflict of Interest

None declared.
